# Age- and sex-related differences in sleep patterns and their relations to self-reported sleep and mood

**DOI:** 10.1093/sleepadvances/zpaf079

**Published:** 2025-11-08

**Authors:** Habiballah Rahimi-Eichi, Justin T Baker, Anders M Fjell, Randy L Buckner

**Affiliations:** Department of Psychology, Center for Brain Science, Harvard University, Cambridge, MA, United States; Institute for Technology in Psychiatry, McLean Hospital, Belmont, MA, United States; Department of Psychiatry, Harvard Medical School, Boston, MA, United States; Institute for Technology in Psychiatry, McLean Hospital, Belmont, MA, United States; Department of Psychiatry, Harvard Medical School, Boston, MA, United States; Center for Lifespan Changes in Brain and Cognition, University of Oslo, Oslo, Norway; Center for Computational Radiology and Artificial Intelligence, Oslo University Hospital, Oslo, Norway; Department of Psychology, Center for Brain Science, Harvard University, Cambridge, MA, United States; Department of Psychiatry, Harvard Medical School, Boston, MA, United States; Athinoula A. Martinos Center for Biomedical Imaging, Massachusetts General Hospital, Boston, Charlestown, MA, United States

**Keywords:** UK Biobank, sleep, aging, actigraphy, depression

## Abstract

Sleep is a fundamental biological process associated with diverse physiological and psychological functions, yet systematic, population-level, objective descriptions of its variation across demographic and psychological factors are still emerging. Here, we characterize age- and sex-related differences in sleep and their associations with mood using week-long actigraphy data from UK Biobank participants aged 44–82. Robust age- and sex-related differences in sleep were identified (*n* = 38 546) and replicated (*n* = 38 547), reflecting reliable nonlinear interactions between age and sex. Younger women slept about 17 min more than their male counterparts, though this difference diminished with age, with both sexes reducing total sleep duration in later life. Middle-aged individuals exhibited shorter sleep durations during the week, with weekend sleep increasing by as much as 50 min. Participants in their seventh and eighth decades showed more consistent sleep patterns throughout the week. Sleep patterns also suggest maintenance of total sleep duration: individuals reporting waking too early maintain sleep duration by going to sleep earlier, while individuals reporting sleeping too much fall asleep later but also wake later, again maintaining sleep duration. Self-reported depression and anhedonia were associated with reduced total sleep duration across multiple age groups and both sexes. By systematically mapping actigraphy-derived sleep features across demographic strata and linking them to subjective reports of sleep and mood, this study provides an integrated framework that complements and extends prior findings, offering a valuable reference point for future investigations of sleep–mood associations in large cohorts.

**Statement of Significance**

Using large-scale actigraphy from the UK Biobank (*n* = 77 093), our study identifies robust, nonlinear interactions between age and sex in shaping objective sleep duration and timing across mid-to-late adulthood. We further link these objective sleep features to self-reported sleep quality and mood disturbances, including depression and anhedonia. Building on prior investigations of similar datasets, our approach emphasizes rigorous quality control, replicability, and a focus on demographic interactions that have received comparatively less attention in sleep research. In doing so, these findings contribute to a more nuanced understanding of how biological and demographic factors jointly influence sleep patterns and their mental health correlates—supporting the broader goal of advancing precision sleep medicine and population-level risk profiling for sleep-related dysfunctions.

## Introduction

Sleep is an essential biological function that supports diverse physiological and psychological processes, including metabolic health, immune system resilience, and cognitive performance [1, 2]. Disruptions in sleep patterns, including reduced sleep efficiency, early morning awakenings, and increased wake after sleep onset (WASO), are frequently observed in neurodegenerative conditions such as Alzheimer’s and Parkinson’s disease [3–5]. Longitudinal and biomarker studies have documented that sleep disturbances commonly accompany these disorders, underscoring their relevance as a characteristic feature of aging-related health profiles. Beyond neurodegeneration, sleep disturbance is increasingly recognized as a transdiagnostic marker that co-occurs with a wide range of mental health conditions across the lifespan, suggesting shared biological and behavioral pathways that shape both sleep and mood regulation [6]. Meta-analytic evidence further indicates that diverse forms of sleep disruption, including insomnia and hypersomnia, are consistently associated with a greater likelihood of first-onset mood and psychotic disorders in youth and early adulthood, highlighting the importance of population-scale characterization of sleep–mental health relationships [7]. It is therefore critical to understand how sleep patterns differ by age and sex and how these patterns relate to mood and well-being. Here, we provide such a normative reference by estimating sleep patterns under natural conditions using objective actigraphy in a large cohort of 77 093 individuals.

While previous research has examined sleep patterns across age and sex, the reliance on self-reported data can introduce bias, particularly among older adults who may have altered self-perceptions of sleep [8–10]. To address these limitations, large-scale studies increasingly use actigraphy, which provides objective measures of sleep and activity in natural settings [11–13]. Although polysomnography (PSG) remains the gold standard, it is impractical for continuous, population-wide monitoring. Despite the advantages of actigraphy, prior work has often emphasized single aspects of sleep (e.g. duration or broad age trends) without systematically examining how demographic factors such as age and sex are associated with multiple dimensions of sleep. Moreover, objective sleep measures are not always considered alongside self-reported experiences, limiting our understanding of how actigraphy-derived patterns correspond to subjective perceptions of sleep and mood disturbances. Opportunities therefore remain to synthesize objective and subjective measures in a structured, comparative framework that can enrich descriptions of sleep variation across the adult lifespan.

In this study, we characterized daily sleep and wake activity in middle-aged and older adults (ages 44–82) using week-long actigraphy data from the UK Biobank. Systematic quality control ensured inclusion of participants with complete, continuous data over 6 days, enabling robust characterization of sleep parameters. Our analyses focused on sleep duration, onset, and wake time, with attention to age- and sex-related differences and the correspondence between objective measures and self-reports. We also examined how mood symptoms, particularly depression and anhedonia, were related to objective sleep measures. To address these questions, we pursued five primary aims: (1) quantify age- and sex-related differences in sleep duration, onset, and wake time; (2) characterize weekday–weekend variation across demographic groups; (3) describe age- and sex-related patterns in daytime wake activity and diurnal rhythms; (4) evaluate the correspondence between objective sleep estimates and self-reported sleep quality, including perceptions of too much sleep or early awakenings; and (5) assess how objective sleep and activity patterns are associated with self-reported mood symptoms. While our results align with prior work linking sleep and mood, they are interpreted as associations rather than directional effects, given the temporal separation between actigraphy and self-reported measures. Together, these aims provide a comprehensive framework for describing how normative sleep and activity patterns vary across the adult lifespan and how they relate to subjective experiences of sleep and mental health.

## Methods

### Participants

Participants were drawn from the UK Biobank, a large-scale cohort of over 500 000 individuals at recruitment, who were assessed at 22 centers across the United Kingdom [14]. This cohort provides a semi-representative sample of the population, encompassing a wide range of sociodemographic, lifestyle, and health characteristics [15]. A subset of 111 635 participants, ages 44–82 years, was randomly selected during 2014–2015 to wear wrist-worn accelerometers for the passive measurement of physical activity levels, after providing informed consent [16]. In 2017, an online follow-up questionnaire focused on mental health, largely based on the World Health Organization Composite International Diagnostic Interview, was completed by approximately 159 000 participants [17]. From this assessment, questions related to sleep, depression, and anhedonia were utilized here to compare self-reports of sleep and mood with objective activity measures. Ethical approval for data re-analysis was granted by the Harvard University Human Studies Committee under UK Biobank Application 67237.

### Estimation of activity levels and major sleep episode from raw accelerometer data

**Figure 1:**
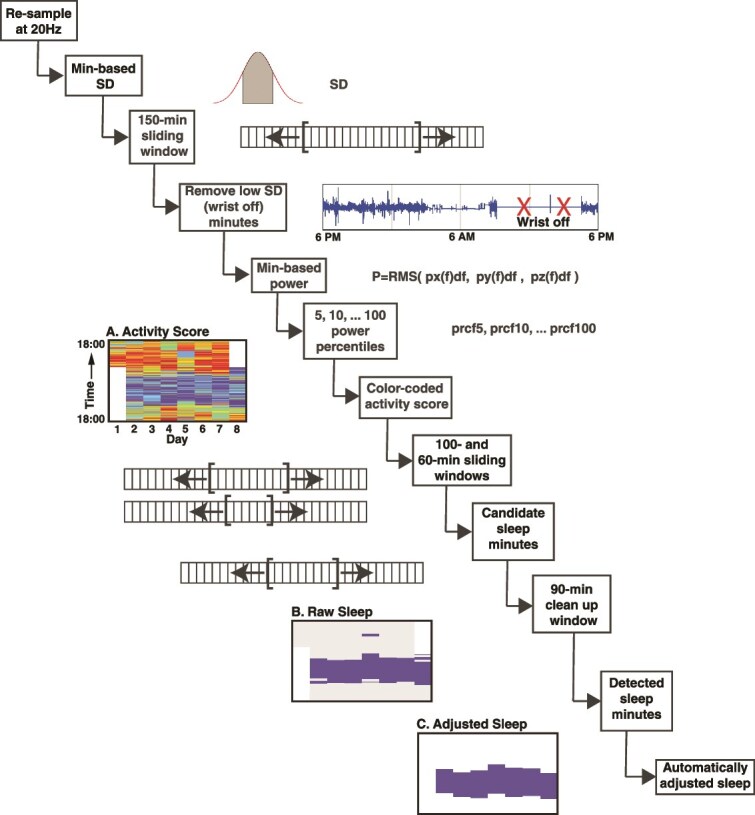
Processing pipeline used to estimate sleep duration. The sequential steps of the fully automated processing pipeline used to estimate the major sleep epoch from the UK Biobank data are illustrated. The pipeline, referred to as DPSleep [18], begins with resampling raw accelerometer data, generating individualized activity scores (A), raw (B), and adjusted sleep episodes (C). Sleep parameters are determined based on automatically adjusted sleep episodes as shown in (C). The raw data are resampled at 20 Hz, and the standard deviations of acceleration across the three axes are averaged over 150-min intervals forward and backward to identify wrist-off minutes. The power density spectrum of the acceleration signal is then computed, and the area under the curve approximates the raw activity power as the root mean square integrated across the axes. Every minute is categorized into percentile thresholds, from 5 to 100, for visualization (A: dark blue to red indicates increasing activity scores) and quantification. Specific thresholds (i.e. 10, 25, 50, and 75 percentiles) are used to determine sleep episodes with a series of forward and backward moving averages applied to identify potential sleep episodes, which are then refined to produce the raw sleep estimate (B) and yield the final adjusted sleep estimate (C).

Accelerometer data were obtained using a three-axis wristband device (model AX3; Axivity Ltd, Newcastle, UK), which recorded participants’ movements continuously for up to 8 days [16]. To derive objective sleep and activity metrics, we applied the open-source DPSleep pipeline [18], modified to handle UK Biobank data (Figure 1). This pipeline utilized the standard deviation of accelerometer signals to detect wrist-off episodes and calculated minute-level power across the three axes using power density spectrum analysis. Within-individual activity levels were classified by percentiles (10th, 25th, 50th, and 75th) using an iterative application of sliding windows (100, 60, and 90 minutes) to robustly estimate the major sleep episode [18]. The longest period of low activity was designated as the sleep episode, without prior assumptions about sleep onset time or duration. Automatic hierarchical rules were applied to refine sleep episode boundaries and merge short adjacent sleep bouts, yielding the final estimate of the major sleep episode. Further details on the validation and limitations of these estimates can be found in Rahimi-Eichi et al. [18].

### Accelerometer data quality control

Accelerometer data from the UK Biobank were first screened to exclude duplicate samples from the same individual, as shown in Supplementary Figure 1. The remaining data were processed using the DPSleep pipeline. After processing, datasets were further excluded if they exhibited structural anomalies or had more than 4 days of missing sleep data. A second round of manual quality control (QC) was then conducted, as depicted in Supplementary Figure 2. This QC process was blinded to participants’ age and sex. Condensed activity plots, displaying daily activity patterns from 200 participants each, were reviewed for missing data or off-wrist periods (Supplementary Figure 2). Off-wrist periods were allowed only on days 1 and 8, provided they did not interfere with the measurement of sleep onset on day 2 or wake time on day 7, ensuring six continuous days of data. Participants whose data coincided with daylight-saving time changes were excluded. These QC procedures ensured inclusion of only high-quality data with sufficient sampling for accurate sleep pattern estimation.

The final resulting sample of 77 093 participants was divided into two equally sized datasets: Discovery (*n* = 38 546) and Replication (*n* = 38 547) (Supplementary Figure 1). These datasets were matched by sex and grouped into age bins of 44–49, 50–54, 55–59, 60–64, 65–69, 70–74, and 75–82 years (Table I). The separation into two datasets allowed for independent replication of findings.

**Table I:** Participant counts: Participant counts after quality control (QC), stratified by age group and sex. Counts are shown alongside the percentage of the total sample (*n* = 77 093)

	44–49	50–54	55–59	60–64	65–69	70–74	75–82	All ages
**XX**	2904 (3.77%)	5909 (7.66%)	7016 (9.10%)	8760 (11.36%)	10 737 (13.93%)	6829 (8.86%)	1249 (1.62%)	43 404 (56.30%)
**XY**	1995 (2.59%)	3854 (5.00%)	4411 (5.72%)	6124 (7.94%)	8952 (11.61%)	6912 (8.97%)	1441 (1.87%)	33 689 (43.70%)

### Self-reported sleep and mood

The UK Biobank online questionnaires included several items assessing mental health and well-being [17]. Participants were asked whether their sleep had changed, with a binary response option (“Yes” or “No”) to the question, “Did your sleep change?” Follow-up questions on “sleeping too much” (Data-Field 20 534) and “waking too early” (Data-Field 20 535) were analyzed to investigate associations between self-reported sleep disturbances and objective sleep measurements. Additionally, during the baseline assessment, participants were asked about their sleep duration through the question, “How many hours of sleep do you get in every 24 hours (please include naps)?” (Data-Field 1160) [14]. Participants could select any integer to respond. We analyzed the association between self-reported sleep duration and objective sleep measurements to ensure consistency and validity.

In the section on depression, participants rated the severity of their symptoms over the past 2 weeks, including feelings of depression (Data-Field 20 510) and anhedonia (lack of pleasure) (Data-Field 20 514). Response options included: “not at all,” “several days,” “more than half the days,” and “nearly every day.” For analysis, responses were dichotomized into two categories: “No” for “not at all,” and “Yes” for any response indicating symptoms for “several days,” “more than half the days,” or “nearly every day.”

### Statistical analysis

The mean and standard error (SE) were calculated to describe the effects of age, sex, and day of the week on sleep parameters. Comparison of self-reported sleep duration groups was conducted using one-way ANOVA; binary self-report groups (i.e. “Yes” vs. “No”) were compared using two-sample *t*-tests, with significance set at *p* < .05 for both tests. In the instances of multiple groups, one-way ANOVAs were utilized with significance set at *p* < .05. Statistical analyses were performed using MATLAB R2022b with the Statistics and Machine Learning Toolbox (MathWorks, Natick, MA, USA). We examined age- and sex-related differences in objective sleep timing and duration (Aim 1), weekday–weekend variation in sleep across age and sex groups (Aim 2), and age- and sex-related differences in daytime wake activity, including overall levels and diurnal patterns (Aim 3). We further assessed associations of objective sleep and activity with self-reported sleep (Aim 4) and with mood symptoms (Aim 5). To evaluate the reproducibility of findings, all analyses were performed independently in two nonoverlapping samples—a Discovery dataset used for primary analyses and a Replication dataset used to confirm key results. The datasets were drawn from distinct participant subsets with no individual overlap and were processed using identical preprocessing and analytical pipelines.

## Results

The results provide a systematic characterization of how objective sleep and activity measures vary by age and sex and how these measures relate to self-reported sleep quality and mental health symptoms. All primary analyses were first performed in a large Discovery dataset and subsequently replicated in an independent Replication dataset to confirm the robustness and generalizability of findings. Each dataset was processed and analyzed separately using identical pipelines, ensuring analytical independence. Moreover, within each dataset, age- and sex-specific groups constitute independent subsamples; thus, convergent findings across groups or between datasets represent genuine replications rather than repeated tests within the same individuals. This design allows observed effects—such as age- and sex-related variation in sleep patterns, weekday–weekend differences, and associations with self-reported outcomes—to be interpreted as consistent and reproducible across independent samples. Together, these analyses offer a comprehensive description of behavioral sleep patterns across the lifespan and reveal consistent associations between objective and self-reported indicators of sleep and well-being.

### Sleep patterns differ by age and sex

**Figure 2:**
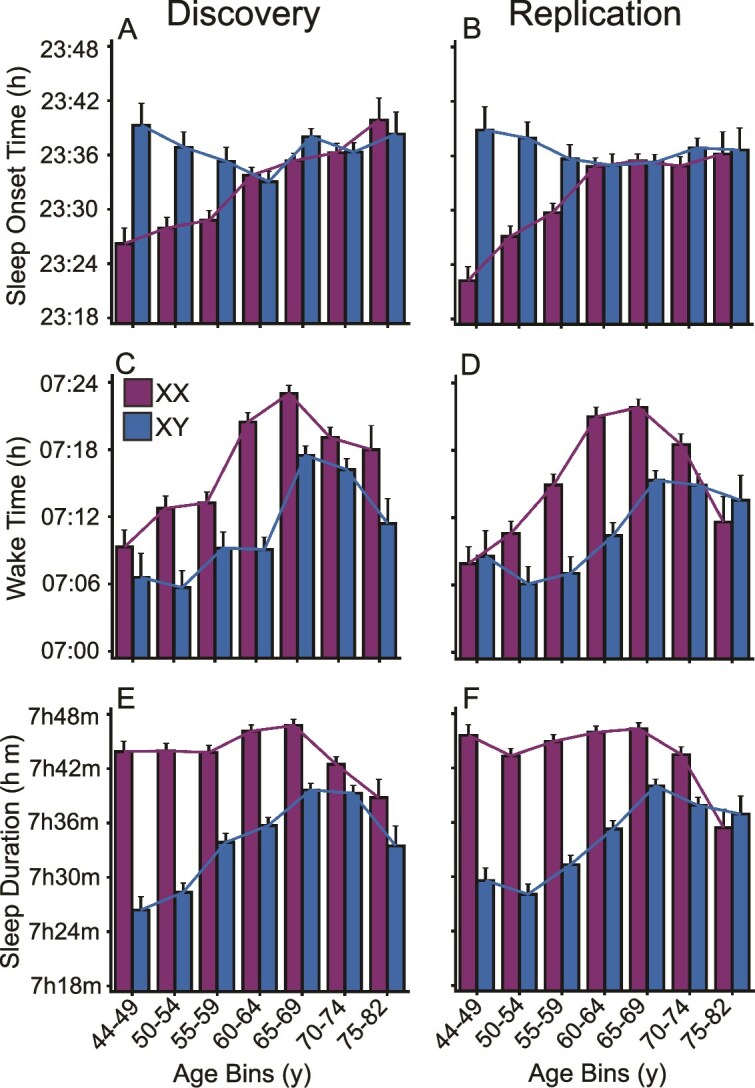
Sleep patterns differ by age and sex. Sleep onset, wake time, and sleep duration are shown for separate age and sex groups. Mean and standard error are displayed for each parameter for each group, where each bar is a distinct group of participants. Here and elsewhere, we scale the plots to capture the data range, which, in some cases, highlights relatively small effects. The left panels (A, C, E) display the sleep patterns for the discovery dataset, and the right panels (B, D, F) the patterns for the independent replication dataset. Men younger than 60 generally go to sleep later (A, B) and wake up earlier than women (C, D), resulting in shorter sleep duration by up to 20 min (E, F). This sex-associated sleep gap diminishes with age. Older men’s sleep durations are longer and older women’s shorter. Older age groups for both sexes tend to wake up later until the 70s, where this trend plateaus or reverses. These idiosyncratic age by sex interaction patterns are robust and replicate across the Discovery and independent Replication datasets. h = hour; y = year.

Replicable differences were observed in sleep duration, sleep onset, and wake time when examined in relation to age and sex. On average, men exhibited shorter sleep durations compared to women across most age groups, particularly in those under 60 (Figure 2, E and F). This difference was accounted for by later sleep onset and earlier wake times in men compared to women. Specifically, men under 60 went to bed later than women, yet woke up earlier, leading to about 17 min in reduced total sleep duration (Figure 2, A and B, Supplementary Table 1). Effect sizes for sleep duration difference between men and women under 60 were small (Cohen’s *d* = 0.22–0.38; 95% CIs [0.18 to 0.25] to [0.32 to 0.44]), yet highly precise given the large sample size. Curve-fitting analyses indicated that, for most sleep parameters, quadratic models provided a better fit (mean *R*^2^ = 0.71 ± 0.23 SD) than linear models (mean *R*^2^ = 0.50 ± 0.40 SD), suggesting nonlinear relationships between age and sleep characteristics.

In the oldest participant groups, the gap between men and women diminished. By the seventh decade of life, men and women demonstrated notably similar sleep durations, and both sexes showed earlier wake times (Figure 2, C and D). This pattern may suggest that the influence of social or occupational factors on sleep duration lessens with age. Given these are cross-sectional group difference estimates, another plausible explanation for this pattern is the higher mortality rate among men in middle age, which could result in the surviving older male sample exhibiting sleep traits more akin to those of women.

Furthermore, in both men and women, the overall trend showed that individuals tended to wake later during their 60s, a trend that stabilized or reversed with increasing age. This observation could reflect a biological shift in sleep–wake regulation during later life stages or changes in lifestyle after retirement.

### Sleep patterns differ between weekdays and weekends in an age- and sex-dependent manner

**Figure 3:**
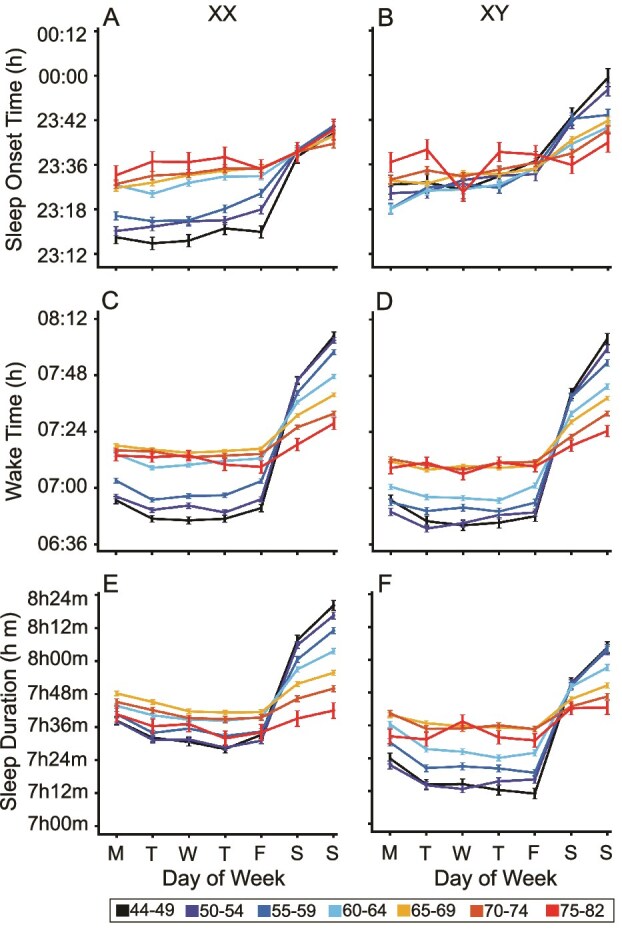
Weekly sleep patterns differ by age and sex. Sleep onset (A, B), wake time (C, D), and sleep duration (E, F) are shown for each day of the week, separated into age and sex groups, for the full data set. Sleep parameters show significant differences between weekdays and weekends for groups under 60 years of age, with later sleep onset and wake times and longer sleep durations on weekends. Male participants younger than 60 show consistent late bedtimes across all days, while participants over 65 exhibit minimal differences between weekday and weekend sleep patterns, achieving balanced sleep duration across the week. XX = genetic female; XY = genetic male. M, T, W, T, F, S, and S indicate the day of the week beginning with Monday. Colors of the lines demarcate the age group, as illustrated by the bottom legend. h = hour.

Participants under 60 showed marked, replicable differences in sleep timing between weekdays and weekends, with later sleep onset, wake times, and about 50 min longer sleep duration on the weekends (Figure 3, E and F). Of further interest, men displayed less variation in sleep onset between weekdays and weekends (Figure 3, B), suggesting that their late bedtimes may not always be linked to work obligations. Results from the Discovery and Replication datasets are provided in Supplementary Figure 3.

For participants over 65, the difference between weekday and weekend sleep patterns diminished, with sleep duration and timing remaining consistent throughout the week. This uniformity may reflect fewer external obligations such as work, which tend to regulate sleep schedules in younger populations. Whatever the origin, these complex, nonlinear patterns are critical to consider in studies of sleep that contain individuals of different ages.

### Wake activity and daily activity patterns differ by age and sex

Wake activity declines with age, with a steeper reduction observed in men compared to women. Supplementary Figure 4 and Supplementary Table 2 illustrate this trend across different age groups and between sexes, as well as across weekdays and weekends. Younger participants, particularly those under 60, exhibited differences between weekdays and weekends, with higher activity reliably observed on weekends in both the independent Discovery and Replication datasets, for both sexes. In contrast, older participants displayed more stable (but lower overall) activity levels across the entire week, showing only slight variations between weekdays and weekends.

To provide a more detailed view, Supplementary Figure 5 presents the average wake activity from 6 pm of the previous day to 6 pm of the current day for all participants, mapped over the days of the week. The Discovery and Replication datasets showed consistent patterns, with higher activity in the mornings and lower activity in the evenings. This daily rhythm was evident across both samples, with a noticeable shift in sleep patterns—particularly wake times—during weekends, where mornings and afternoons were more active.

**Figure 4:**
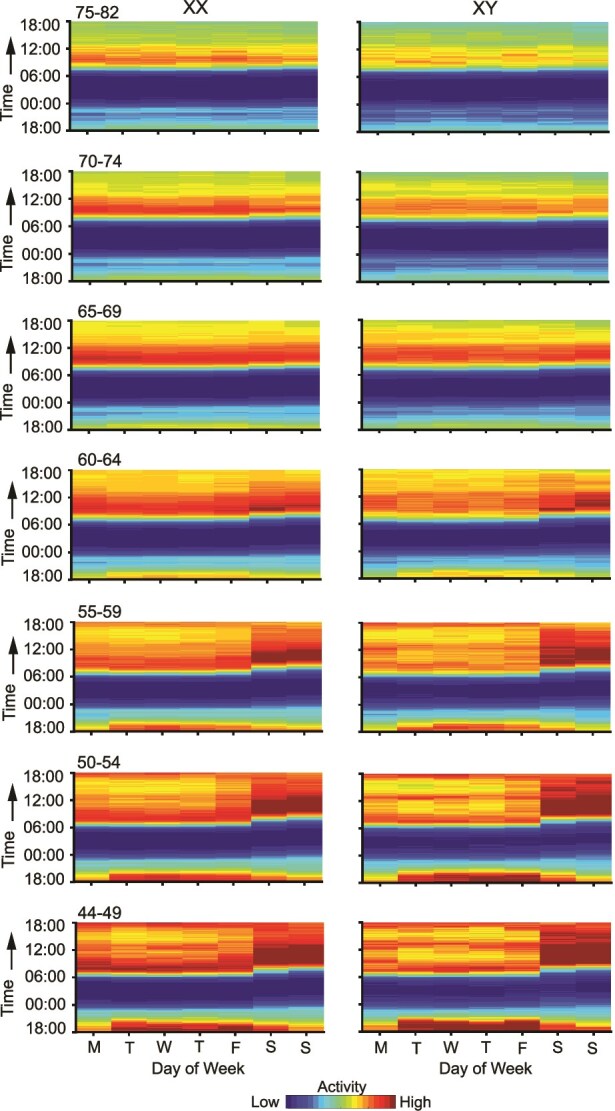
Daily activity maps split by age and sex. Mean raw daily activity for participants is displayed by sex and age group for each day of the week. The maps reveal a marked difference (decline) in activity with age for both sexes, with fewer high-activity periods (red) and more low-activity periods (green) in the oldest groups. Differences in activity levels between weekdays and weekends diminish with age. Morning activity remains higher across all groups with lower evening activity. Weekends are less active in older participants. XX = genetic female; XY = genetic male. M, T, W, T, F, S, and S indicate the day of the week beginning with Monday. Colors range from dark blue to dark red, scaled to the average activity levels across all participants using a fixed color scale as in Supplementary Figure 4.

Figure 4 further breaks down the daily activity patterns by age and sex, using the fixed color scale from Supplementary Figure 5. The plots illustrate a decline in activity with age in both men and women, as indicated by the reduction in higher activity (red) and the increased presence of lower activity (green). In older age groups, the distinction between activity levels during weekdays and weekends diminishes. Additionally, activity was consistently higher in the morning and lower in the evening across all age and sex groups. Notably, weekends became less active on average in the oldest participant groups.

### Objective sleep and activity differ in relation to self-reported sleep

**Figure 5:**
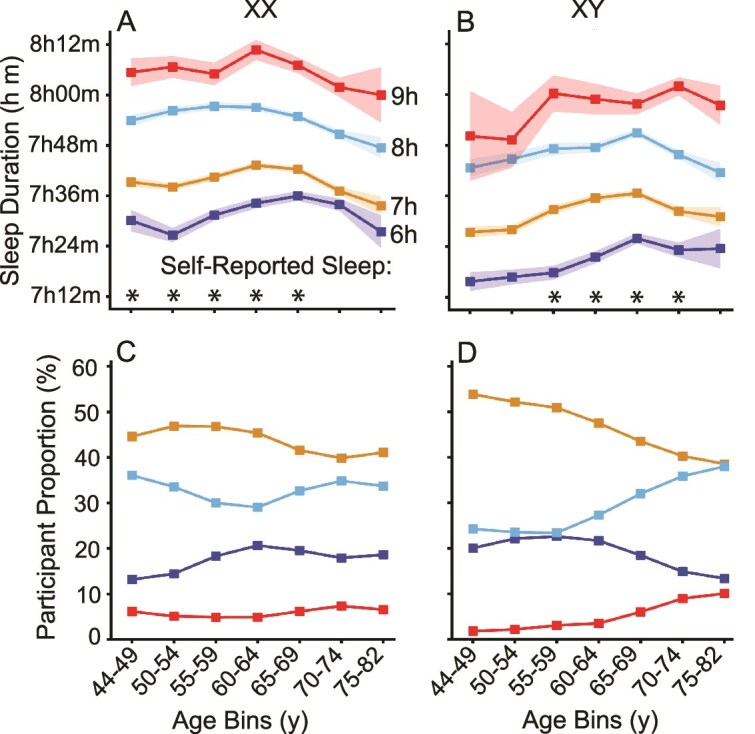
Objective sleep duration for individuals self-reporting their daily sleep duration. Objective sleep duration is shown for participants split by groups with distinct self-reported sleep duration. Colors represent different reported sleep durations from 6 hours to 9 hours. The objective sleep duration differentiates these groups across all ages for both men and women (A, B), though the range for objective sleep duration was between 7 and 8 hours. This is a compression relative to the self-reported duration, and the plotting scale reflects this compression. The proportion of participants in each age group is plotted separately for each sex and color-coded according to the corresponding self-reported sleep duration groups (C, D). Significance was tested using one-way ANOVA (* = *p* < .05).

The objective sleep measures were examined for separate samples based on self-report. As a first validity test, self-reported sleep duration was plotted in relation to objective sleep duration (Figure 5). Participants were categorized into four groups based on their self-reported sleep duration, ranging from 6 to 9 hours. Objective sleep duration was found to parametrically vary as a function of self-reported duration, consistently across age and sex groups (Figure 5, A and B). One-way ANOVA confirmed that the differences in objective sleep duration across the self-reported categories were independently statistically significant in many of the age groups (nine independent between-group contrasts showed a significant effect, *p* < .05). In the remaining five between-group comparisons, the pattern was in the same direction without exception.

The objective measures of sleep duration, though, did not span the same large range as the self-reported differences falling within the 7- to 8-hour range. The proportion of participants in each age group is visualized separately for each sex and color-coded by self-reported sleep duration categories (Figure 5, C and D). Among men, the proportion reporting longer sleep durations (i.e. 8 and 9 hours) increased with age, while the proportion reporting shorter durations (i.e. 6 hours) decreased—a trend not as pronounced in women.

**Figure 6:**
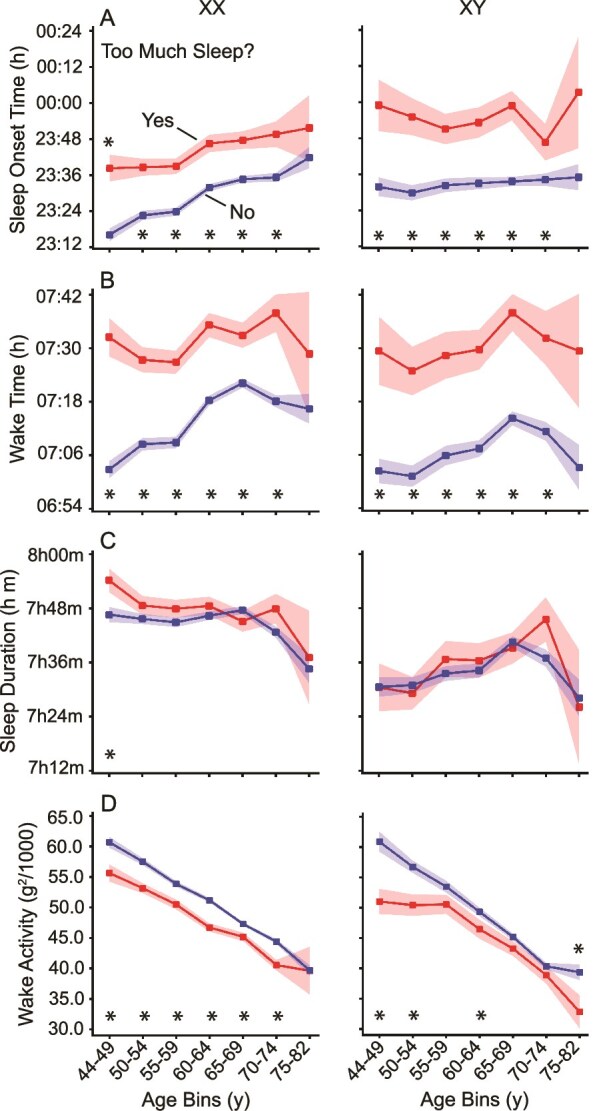
Sleep and activity parameters for individuals self-reporting “too much sleep.” Sleep parameters and daily activity means are shown for participants responding “yes” or “no” to whether they were “sleeping too much.” Yes indicates those reporting too much sleep, and No indicates the others, both with standard error bars. Participants reporting excess sleep wake up (B) and go to sleep (A) later, though their sleep duration does not significantly differ (C). They also show lower daily activity (D), possibly due to reduced overall activity or increased daytime napping. Significance was tested using a two-sample *t*-test (* = *p* < .05).

The relationship between objective sleep measures and self-reported sleep disturbances, such as “too much sleep” or “waking too early,” was also examined. Participants who reported “too much sleep” exhibited later sleep onset and wake times (Figure 6, A and B), but their total sleep duration was not significantly greater than those who did not report oversleeping (Figure 6, C). Interestingly, participants reporting excessive sleep tended to have lower wake activity during the day (Figure 6, D), suggesting that they may be less physically active.

**Figure 7:**
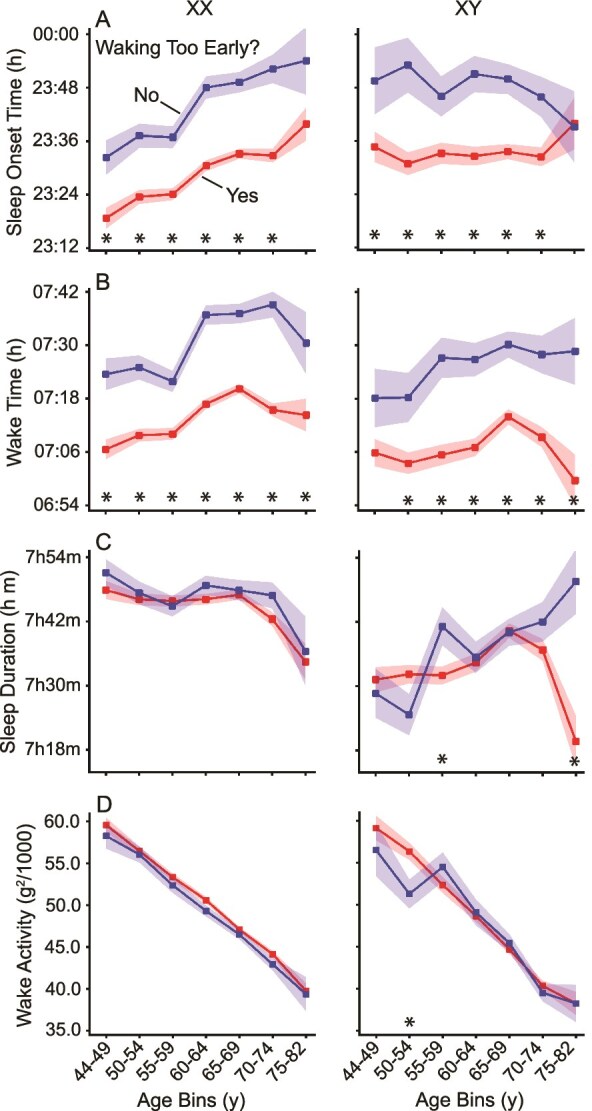
Sleep and activity parameters for individuals self-reporting “waking too early.” Sleep parameters and daily activity means are shown for participants who responded “yes” or “no” to whether they were “waking too early.” Those who reported waking early (Yes) and those who did not (No) are shown with standard error bars. Those self-reporting waking too early go to sleep earlier (A), resulting in similar sleep duration (C) to those responding no. Daily wake activity levels are comparable between groups (D). Significance was tested using a two-sample *t*-test (* = *p* < .05).

Participants who reported “waking too early” had earlier sleep onset and wake times compared to those without early awakenings (Figure 7, A and B). However, again, their total sleep duration was similar to the nonearly-waking group (Figure 7, C), indicating that while they woke earlier, they also went to bed earlier. This finding suggests that early waking may not necessarily indicate reduced sleep duration but rather an adjustment in sleep timing.

### Objective sleep and activity differ in relation to self-reported mood

**Figure 8:**
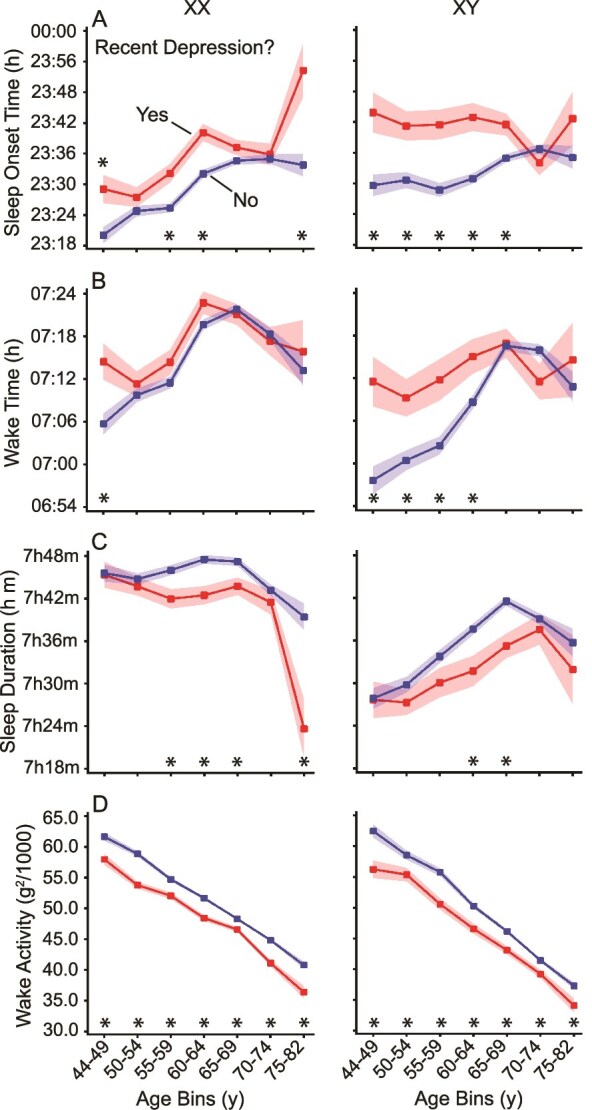
Sleep and activity parameters for individuals with recent self-reported symptoms of depression. Sleep parameters and mean wake activity are shown for participants responding “no” (not at all) or “yes” (any frequency) to feeling down, depressed, or hopeless over the prior 2 weeks. Individuals with recent depressive symptoms (Yes) and without (No) are shown with standard error bars. Participants with symptoms of depression go to sleep slightly later (A) with slightly shorter sleep duration (C). Symptoms of depression are associated with notably lower wake activity compared to other participants across ages (D). Significance was tested using a two-sample t-test (* = *p* < .05).

Participants who reported recent depression (Figure 8) or anhedonia (Supplementary Figure 6) exhibited consistently lower daily wake activity compared to those without these symptoms (Figure 8, D, Supplementary Figure 6, D), across ages and both sexes. Recently depressed older women also showed reduced sleep duration, though this difference was not always present (Figure 8, C). No significant duration relationships were observed before the age of 54. An association between late sleep onset and depressive symptoms was particularly pronounced in men (Figure 8, B), underscoring the relevance of sleep timing for mental health.

## Discussion

Age- and sex-related differences in sleep were found and replicated across a large sample of 77 093 participants ages 44 to 82 years from the UK Biobank. Through rigorous quality control of actigraphy data, we identified complex patterns in sleep onset, wake time, and sleep duration that suggest reliable nonlinear interactions between age and sex. Of particular interest are differences between women and men that attenuate in the oldest individuals. We also observed shifts in sleep and wake time linked to self-reported sleep behavior that did not associate with differences in overall sleep duration. By making these observations in a large sample with confidence, we contribute to a growing literature on sleep, aging, and mental health. The complex relations between self-reported sleep and the objective measures further underscore the importance of objective sleep measures in understanding age- and sex-specific sleep patterns. Our analyses provide a population-level normative reference for how objectively measured sleep patterns vary across age and sex and how these patterns correspond to self-reported sleep and mood measures. Rather than inferring mechanisms, these findings establish robust correlational benchmarks for future mechanistic and longitudinal studies.

### Sleep patterns reveal reliable nonlinear interactions with age and sex

Men exhibited shorter sleep durations compared to women, especially in the middle-aged groups, where men tended to go to sleep later and wake up earlier than women. This pattern aligns with previous findings that suggest men sleep less, which could be due to occupational, social, or biological factors. These differences may be associated with men’s tendency to view sleep as an “unfortunate necessity” that competes with work responsibilities, alongside workplace cultures that often value long working hours, though the relationship between these factors and sleep duration remains complex, and also these factors, of course, apply to both sexes to varying degrees [19, 20]. Of interest to the study of aging, in older men and women, this sleep difference narrowed, with both sexes achieving more similar sleep durations. Specifically, men’s sleep duration was increased in older age, except in the very oldest individuals, while women’s sleep duration was higher but then shortened in the eighth decade (Figure 2), a finding consistent with research that shows age-related differences in sleep are more pronounced in men [21]. The observed sex differences in sleep may also relate to hormonal variations across the female reproductive lifespan. The menstrual cycle has been linked to changes in spindle activity and reduced sleep quality around menstruation [22], while the menopausal transition is associated with estrogen withdrawal and increased nocturnal awakenings, independent of vasomotor symptoms [23]. These associations are likely multifactorial, reflecting both biological and psychosocial influences.

Prior work using UK Biobank data reported similar patterns, showing that women tended to sleep longer than men and that sleep–mortality associations were consistent across sex and age groups, although that study did not focus on how sleep duration itself changes with age [24]. Supporting our findings, Willetts et al. [25] reported that in the same dataset, men slept slightly less than women and older adults spent a greater proportion of time asleep than younger adults. Zhu et al. [26] similarly reported, using UK Biobank actigraphy data, that women had longer and more efficient sleep than men, and that shorter sleep was more common among older adults. While their work provided valuable large-scale descriptions of sleep patterns, it did not specifically address whether sex differences in sleep duration change across age.

### Sleep patterns differ across the week in an age-dependent manner

For individuals under 60, significant differences in sleep timing were observed between weekdays and weekends, with later sleep onset and wake times on weekends (Figure 3). These findings are consistent with those of Kelly et al. [27], who reported an average weekend–weekday wake time gap of approximately 36 minutes, with more pronounced differences in younger age groups that diminish in older adults, and with Willetts et al. [25], who also observed longer sleep on weekends compared to weekdays. This robust effect has multiple possible causes. One possible explanation for this pattern is social jetlag, a phenomenon in which individuals accumulate sleep debt during the workweek due to work or social obligations and then compensate by sleeping longer on weekends [28, 29]. Social jetlag may also reflect the expression of late chronotypes’ natural circadian preferences when external constraints are relaxed on weekends. Another possibility is that these patterns reflect a preference for sleeping longer on weekends, not solely as a means of recovering sleep debt but as a form of leisure or enjoyment [30]. In our study, men exhibited less pronounced weekend differences in terms of sleep onset, as their sleep onset times were consistently later even on weekdays. This behavior might indicate a prioritization of nighttime leisure activities over sleep, aligning with previous findings that link late bedtimes to recreational or social preferences [20].

Future work will be required to determine the mechanisms behind these clear and reliable shifts in sleep onset and wake times that differ across the week. The presence of sleep differences during the week in younger populations raises concerns about its potential long-term effects on health, as chronic sleep deprivation has been linked to a variety of health problems, including metabolic disorders and cardiovascular disease [28, 31, 32], although the relation of sleep duration and health consequences is nuanced [33]. Recently, Chaput et al. [34] found weekend catch-up sleep to be common in the UK Biobank but unrelated to mortality or cardiovascular disease; age and sex were considered as covariates but not in relation to how weekend sleep patterns differ across groups.

Reflecting a robust age difference, older participants (those over 65 years) showed largely uniform sleep patterns throughout the week, possibly because their sleep is less constrained by obligations such as work that vary across days of the week. This reduction in the weekday/weekend gap with age has been observed in previous studies, where older adults tend to exhibit more stable sleep schedules due to retirement or fewer external time pressures [35]. What is notable in the present data is the contrast of the weekly sleep patterns between the middle-aged and the oldest individuals (Figure 3). Consistent with prior work, we also observed that wake activity was lower in older adults, with men showing steeper reductions than women [36]. Younger participants showed clearer differences between weekdays and weekends, whereas older participants displayed more uniform, but overall lower, activity levels across the week.

### Self-reported sleep and mental health associated with objective sleep patterns

Self-reported sleep and mental health symptoms, including depression and anhedonia, were reliably associated with objective sleep parameters. As a first result, speaking to the validity of self-report measures, we initially explored and found that self-reported sleep duration parametrically predicted objectively measured sleep duration. While expected, the consistency of the pattern across 14 independent groups of participants, each revealing a parametric relation among all four levels of the self-report variable, was nonetheless pronounced (Figure 5).

Specifically, individuals self-reporting shorter sleep duration objectively slept less than those individuals reporting longer sleep duration. Revealing a difference between self-report and objective measures, the relation was compressed in that subjective estimates of short versus long sleep duration were considerably larger than the objectively measured differences, but the relationship was robust and existed for both men and women across all age groups. These findings support the utility of self-reported measures for assessing sleep behaviors while also highlighting that subjective estimates can diverge systematically from objective measures, potentially reflecting differences in perceived sleep quality or time spent trying to fall asleep. Consistent with prior validation studies showing only moderate correspondence among actigraphy, polysomnography, and self-reports [37], our results underscore the importance of integrating both modalities to capture complementary aspects of sleep behavior. Similar to observations in clinical populations where subjective sleep quality improved despite persistently low actigraphy indices [38], our data indicate that perceived and measured sleep represent related but distinct constructs that may diverge further with age or health status. In UK Biobank data, Zhu et al. [26] contrasted objective actigraphy estimates with previously collected self-reported sleep duration; however, their analysis focused on sociodemographic and lifestyle correlates and did not model age-by-sex interactions. The compression of differences may potentially also result from the lower reliability of the self-reported durations. Additionally, differences in the distribution of self-reported sleep durations by sex and age help explain why younger men exhibit shorter objective sleep durations; a larger proportion of younger men reported shorter sleep (e.g. 6 hours), while reports of longer sleep (e.g. 8–9 hours) became more common with age—a shift not observed in women.

A further interesting finding was that self-report of “waking too early” was consistently associated with earlier objective wake times, as well as proportionate shifts in evening sleep onset times (Figure 7). As a result, total sleep duration was broadly similar regardless of self-report of wake time. This result is consistent with the self-report, which here focused specifically on wake time, but does indicate that endorsing “waking too early” is not a proxy for total sleep duration. Even more striking was that endorsing “too much sleep,” a question that on the surface targets a construct expected to relate to sleep duration, was also not associated with an objective difference in total sleep duration. Rather, like endorsing early rising, self-report of “too much sleep” was associated with a shift in sleep–wake times with those reporting sleeping too much falling asleep and waking later than those who did not make such an endorsement. Furthermore, endorsing “too much sleep” was consistently linked to lower wake activity in both sexes across almost all age groups. Such an effect on activity was absent in those endorsing “waking too early.” These results indicate that self-reported sleep measures are capturing reliable differences in objective sleep parameters but not in a simple or straightforward manner that could be intuited without the available objective sleep measures.

Of most interest, individuals reporting recent depression (Figure 8) or anhedonia (Supplementary Figure 5) exhibited later sleep onset times and shorter sleep durations compared to those who did not endorse depressive symptoms. The effect was not observed in every group but was present and independently significant (replicated) in multiple groups across the age range including both sexes. These findings support the established link between sleep disturbances and depression [39–41]. In a large longitudinal analysis of self-reported sleep data from the UK Biobank, Fatima et al. [41] identified stable sleep trajectories over several years, showing that individuals with persistent depressive symptoms were more likely to remain in poor-sleep groups, suggesting that mood-related sleep disturbances are chronic rather than transient. Using self-reported measures of sleep quality and duration, Hu et al. [39] found that depression partly mediated the relationship between poor sleep and reduced health-related quality of life in older adults. Wainberg et al. [40] employed objective accelerometry-based sleep measures and demonstrated that shorter, less efficient, and more variable sleep patterns were consistently associated with lifetime psychiatric diagnoses, particularly major depression. Together, these studies and our present results converge to show that both self-reported and objectively measured sleep variation are closely linked to depressive symptoms across timescales and clinical boundaries. Interestingly, while depression was associated with reduced sleep duration, it was also linked to reduced wake activity, further suggesting that individuals with low mood may engage in less physical activity throughout the day, a pattern consistent with previous studies [42].

A bidirectional relationship between sleep and depression is well documented. Changes in sleep duration or timing can exacerbate mood disorders, while depression can lead to changes in sleep duration and timing [43]. Our results corroborate the complex interaction between sleep and mood, particularly the association between sleep timing and mental health symptoms. Individuals with late sleep onset and late wake times, especially men, were more likely to report experiencing symptoms of depression, emphasizing the possible association of chronotypes with mental health. Prior research has shown that earlier sleep times are associated with a lower risk of depression, potentially due to greater exposure to daylight, which has mood-enhancing effects [44]. Importantly, however, our findings should not be interpreted as evidence of a bidirectional relationship, given the temporal separation between the actigraphy and self-reported measures. While our results do not resolve the mechanism of the association between sleep parameters and symptoms of depression, the results illustrate that the relationship is present across many cohorts that vary by sex and age.

### Limitations

While actigraphy is a valuable tool for large-scale, real-world sleep assessment, it does not provide the detailed sleep stage information available through PSG. PSG, as the gold standard for identifying specific sleep disorders and sleep stages, is not well suited for large-scale studies due to the burden and cost. Further, PSG necessarily disturbs normal sleep patterns and is not suited to measure natural variations in sleep duration. Advances in actigraphy technology, including the integration of heart rate and heart rate variability measurements, may enhance their ability to estimate sleep stages. Ambulatory PSG may also become viable at scale in the future.

Additionally, the UK Biobank cohort includes cross-sectional participants ages 44 and older, limiting the ability to chart patterns in younger adults and to explicitly estimate how sleep patterns change with age [45]. Given the cross-sectional design and temporal separation between objective and self-reported measures, all reported relationships are correlational and do not imply causation. Furthermore, the limited ethnic diversity of UK Biobank participants means that different population compositions or sociocultural contexts might yield other results [46]. Early sleep disturbances in younger cohorts may be particularly informative for understanding risk pathways to mental health and neurodegenerative conditions. While longitudinal research has shown that sleep disturbances accompany biomarker changes in Alzheimer’s disease and precede Parkinson’s disease onset [47, 48], proposed mechanistic models, such as impaired glymphatic amyloid-beta clearance [49], remain to be tested alongside population-based genetic findings that show little evidence for direct causal effects of sleep traits on these diseases [50, 51]. Future longitudinal and multimodal studies integrating actigraphy with imaging and molecular biomarkers could clarify how sleep characteristics correspond to neurobiological changes across aging. While our analyses characterize age- and sex-related associations between sleep and mood in a nonclinical population, we did not examine clinical diagnoses. Unlike Wainberg et al. [40], who analyzed objective sleep patterns across psychiatric disorders, our design focuses on normative, correlational variation, which limits direct clinical comparability but enhances generalizability to the broader population. Cohort effects, such as differential survivorship among the oldest participants, could also be addressed through longitudinal designs. Extending such analyses to younger populations will be essential for understanding how sleep patterns relate to the development of illness and symptom trajectories over time. Moreover, future research should aim to disentangle sex-related biological factors from gender-related sociocultural influences. Current approaches often conflate these dimensions, limiting interpretability. Developing frameworks that capture both biological variability and gendered experiences will be essential for a more precise and equitable understanding of the observed effects.

### Future directions and implications

In addition to the findings themselves, the present results underscore the importance of age- and sex-specific analyses to understand sleep patterns. An immediate implication of our results is that non-linear sleep patterns and interactions should be considered when exploring the relation of sleep and other factors. For example, regressing the linear effects of age or the age × sex interaction may be insufficient to capture the complex patterns observed here. The present normative description of age and sex differences in sleep patterns is thus valuable as a reference to guide future study designs and analyses that target older populations.

The variation in sleep patterns between groups and individuals within the groups was marked. Finding reproducible relations between self-reported perceptions of sleep and mood indicates that the variation contains a structured signal, not happenstance noise. However, the present results do not provide insights into the biological or other factors that cause the individual differences. Future research, including using the UK Biobank data itself, might explore integrating genetic factors and neuroimaging to examine how sleep patterns are influenced by genetic predispositions and associated with variations in brain structure. For instance, specific genetic polymorphisms related to circadian rhythm regulation and sleep architecture could interact with age- and sex-related changes in sleep [52]. Combining actigraphy data with genotyping could potentially help identify genetic markers associated with sleep resilience or susceptibility to sleep disturbances, which may be predictive of cognitive decline [53, 54]. The present normative characterization of sleep patterns and how they differ by age and sex provides a needed foundation for such future research.

A further intriguing possibility along these lines is that some of the genetic variations associated with sleep patterns or sleep quality in early life may be risk factors for brain aging and dementia later in life because they are linked to broad sequelae that affect the brain and body. That is, some of the genetic variation associated with dementia risk may be related to mechanistic links involving sleep that are associated with health throughout life and may indirectly reflect risk for cognitive decline.

Another future direction will be to explore the association of sleep with brain aging. Sleep pattern differences that vary by age and sex could inform structural brain differences and longitudinal change. Although prior neuroimaging studies have suggested links between poor sleep and brain atrophy, recent large-scale analyses challenge this view [55], highlighting the need for future research to disentangle sleep-related correlates from causal effects on brain structure. Longitudinal studies incorporating structural MRI could be undertaken to examine how age-related sleep differences correlate with brain atrophy and effects on white matter integrity [56].

## Conclusion

In summary, this large-scale actigraphy study of more than 77 000 adults from the UK Biobank provides a comprehensive, normative reference for how objectively measured sleep and activity patterns vary with age and sex and how these patterns correspond to self-reported sleep and mood. The results reveal consistent, replicable demographic differences—particularly nonlinear interactions between age and sex in sleep duration and timing—and demonstrate that subjective perceptions of sleep and mood map onto objective sleep measures in structured but non-equivalent ways. These findings highlight the importance of considering demographic context in interpreting sleep metrics and underscore the need for integrated models that incorporate both objective and self-reported data. While our analyses are correlational, they establish a foundation for future longitudinal and multimodal studies that can clarify the biological and environmental mechanisms underlying age- and sex-related variations in sleep and their implications for mental health and neurodegenerative risk.

## Supplementary Material

RahimiEichi_etal_UKBSleep_251102_SUPP_zpaf079

## Data Availability

This research was conducted using data from the UK Biobank under application number 67237. The UK Biobank data are available to bona fide researchers upon application at https://www.ukbiobank.ac.uk/register-apply.
