# Supplementary material for: Age- and sex-related differences in sleep patterns and their relations to self-reported sleep and mood
Source: Sleep Adv. 2025 Nov 8;6(4):zpaf079. doi: 10.1093/sleepadvances/zpaf079 (PMC12667273; doi:10.1093/sleepadvances/zpaf079)
Supplement: RahimiEichi_etal_UKBSleep_251102_SUPP_zpaf079 [file rahimieichi_etal_ukbsleep_251102_supp_zpaf079.pdf]

Supplemental Figures and Tables

Age- and Sex-Related Differences in Sleep Patterns and Their Relations to Self-Reported Sleep and Mood

Habiballah Rahimi-Eichi[a,b,c], Justin T. Baker[b,c], Anders M. Fjell[d,e] , and Randy L. Buckner[a,c,f]

[a] Department of Psychology, Center for Brain Science, Harvard University, Cambridge, MA, USA; [b] Institute for Technology in Psychiatry, McLean Hospital, Belmont, MA, USA; [c] Department of Psychiatry, Harvard Medical School, Boston, MA, USA; [d] Center for Lifespan Changes in Brain and Cognition, University of Oslo, Oslo, Norway; [e] Center for Computational Radiology and Artificial Intelligence, Oslo University Hospital, Oslo, Norway; [f] Athinoula A. Martinos Center for Biomedical Imaging, Massachusetts General Hospital, Boston, Charlestown, MA, USA

Correspondence should be addressed to Dr. Habiballah Rahimi-Eichi, Email: hrahimieichi@mgb.org

This PDF file includes:
Supplemental Figures 1 to 6
and **Supplemental Tables I and II**

This PDF document includes Supplemental Figures 1 and 2, corresponding to the Methods section, as well as Supplemental Figures 3–5, which present the discovery and replication plots. Supplemental Figure 6 displays the results related to mental health (anhedonia). Additionally, the document contains two supplemental tables summarizing the t-test results for sex differences in sleep and activity parameters.

Supp. Figure 1. Flow chart of participant inclusion. A flow chart illustrates the participant selection that resulted in two independent Discovery and Replication datasets used for all subsequent analyses. Out of 111,625 initial candidate samples exclusions included repeat samples, wrong data structure, no sleep detected, missing data, manual QC failures (see Supp. Figure 2), and exclusion for daylight savings time. These exclusions were all made before examining sleep structure to mitigate bias. The final sample included 77,093 participants, divided into two equal groups for Discovery (N=38,546) and Replication (N=38,547). To visualize effects of age and sex the data were further separated into age bins (44-49, 50-54, 55-59, 60-64, 65-69, 70-74, 75-82) and separated groups by genetic sex (XX = genetic female; XY = genetic male).

Supp. Figure 2. Manual quality control. To ensure only high-quality data was included in subsequent analyses, a manual quality control (QC) procedure was developed that could efficiently accommodate the large sample size. Exclusions were made blind to age or any other features of the data to minimize bias. Every sample was visualized as a week-long panel that showed the day-by-day estimated activity. A usable example (A) with complete actigraphy data for 6 consecutive nights and days is shown alongside an unusable example (B) with several missing days, indicated by flat or low-variance activity scores (blue). QC maps (C) display activity scores created for 200 participants per page, allowing review of every sample. Only individuals with complete sleep and wake accelerometer data for days 2 though 7 were included in the analysis (days 1 and 8 were often truncated, as expected, reflecting watch wearing began and ended).

Supp. Figure 3. Weekly sleep patterns: Discovery and Replication. Sleep onset, wake time, and sleep duration are shown for each day of the week, separated into age and sex groups, similar to Figure 3. Plots are replicated for the Discovery (top panels)) and independent Replication (bottom panels) datasets. XX = genetic female; XY = genetic male. M, T, W, T, F, S, S indicates day of the week beginning with Monday. Colors of the lines demarcate the age group, as illustrated by the bottom legend. h = hour.

Supp. Figure 4. Wake activity patterns differ by age. Mean raw activity during wake episodes is shown separated by sex and age group for each day of the week. Wake activity differs (declines) significantly with age, with a slightly steeper decline observed in men. Younger participants exhibit more variation in wake activity between weekdays and weekends, particularly among men, whereas older participants maintain stable activity levels across the week. Plots are replicated for the Discovery (top panels)) and independent Replication (bottom

panels) datasets. XX = genetic female; XY = genetic male. M, T, W, T, F, S, S indicates day of the week beginning with Monday. Colors of the lines demarcate the age group, as illustrated by the bottom legend.

Supp. Figure 5. Daily activity maps split by day of the week. Mean raw daily activity from 6 PM on the previous day to 6 PM on each day is mapped across days of the week for Discovery, Replication, and the combined (All) datasets. All data are combined across age and sex. Both Discovery (top panel) and Replication (middle panel) datasets show similar patterns with higher morning activity and lower evening activity. Sleep pattern shifts, particularly in wake times during weekends, are observed consistently across samples, with more active mornings and afternoons on weekends. Colors range from dark blue to dark red, scaled to the average activity levels across all participants.

Supp. Figure 6. Sleep and activity parameters for individuals with recent self-reported anhedonia. Sleep parameters and daily activity means are shown for participants who responded "no" (not at all) or "yes" (any frequency) to experiencing little interest or pleasure in activities over the prior two weeks. Individuals with anhedonia (red) and without (blue) are shown with standard error bars. Participants with anhedonia go to sleep later (A) and have slightly shorter sleep duration (C). The group reporting anhedonia exhibits lower wake activity compared to other individuals across all ages (D). Significance was tested using a two-sample t-test (* = $p < 0.05$).

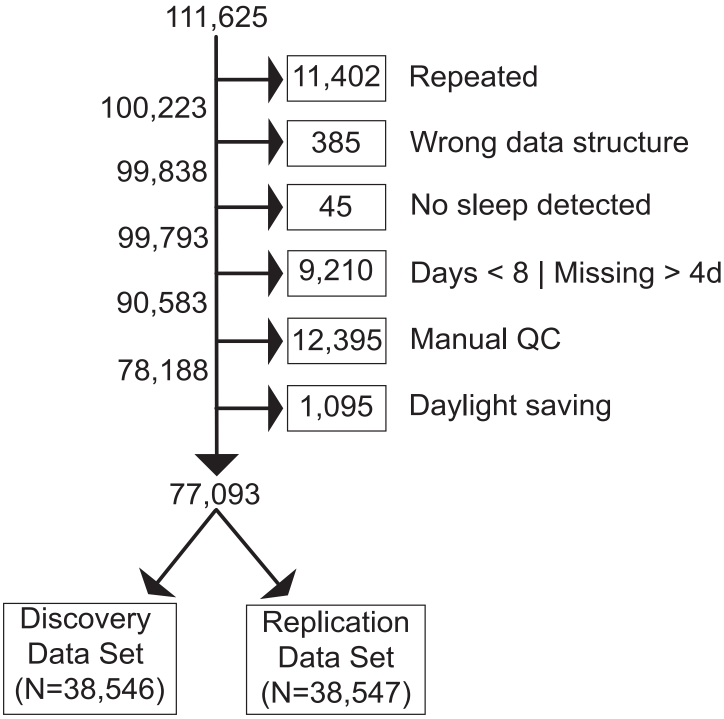

Supp. Figure 1

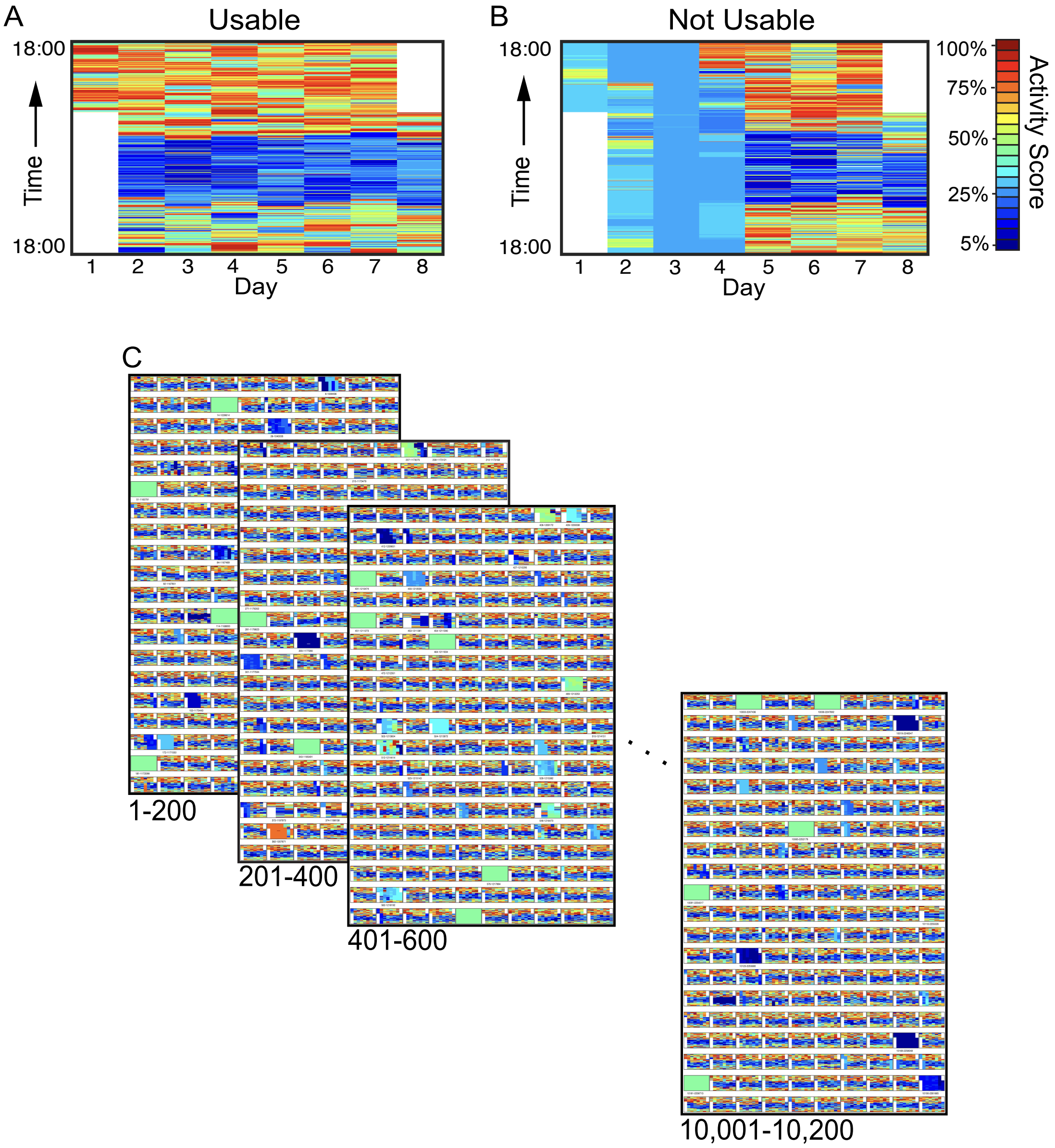

Supp. Figure 2

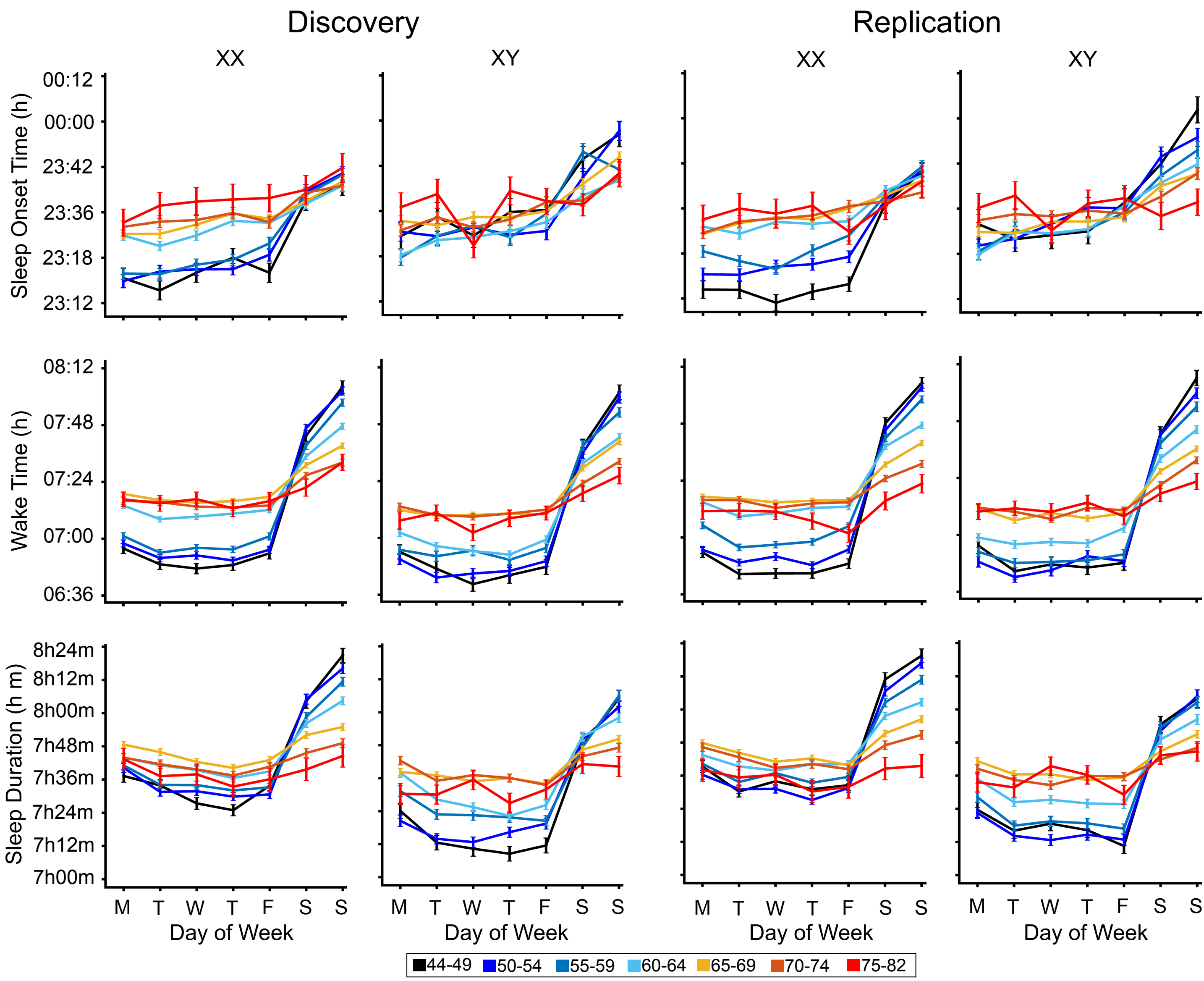

Supp. Figure 3

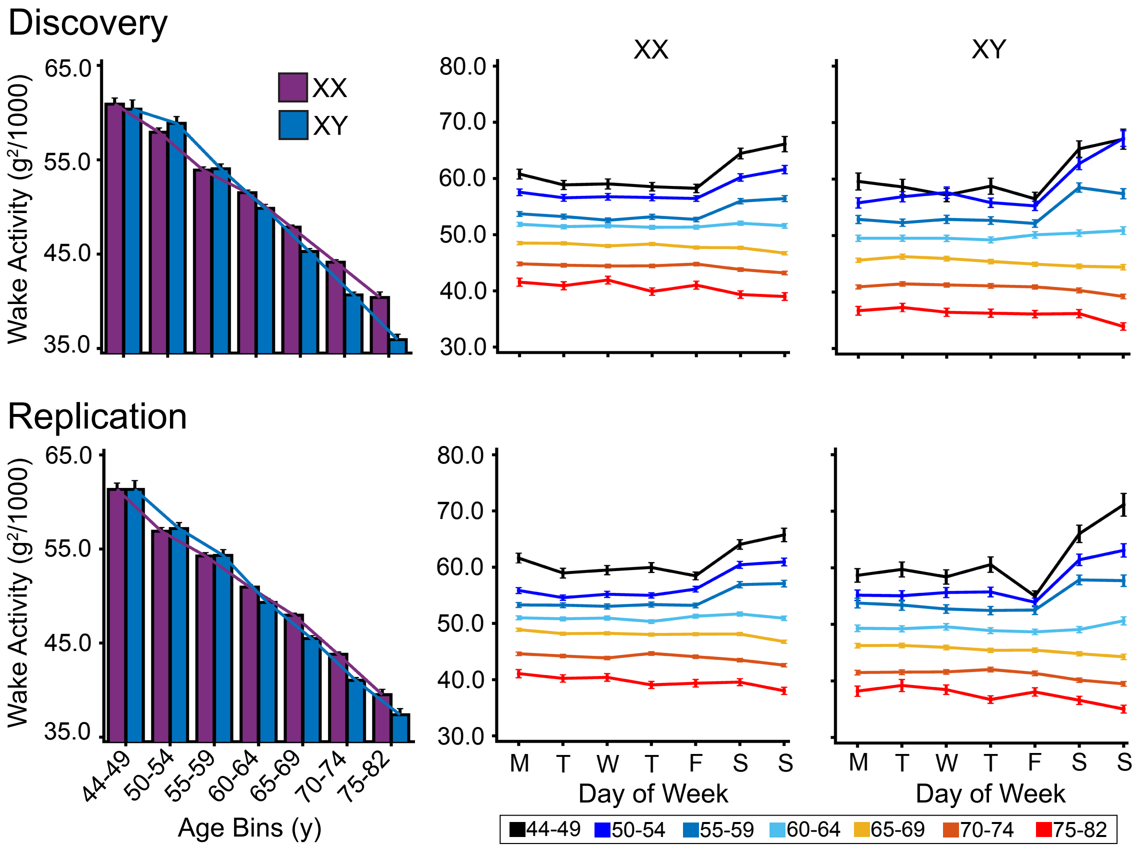

Supp. Figure 4

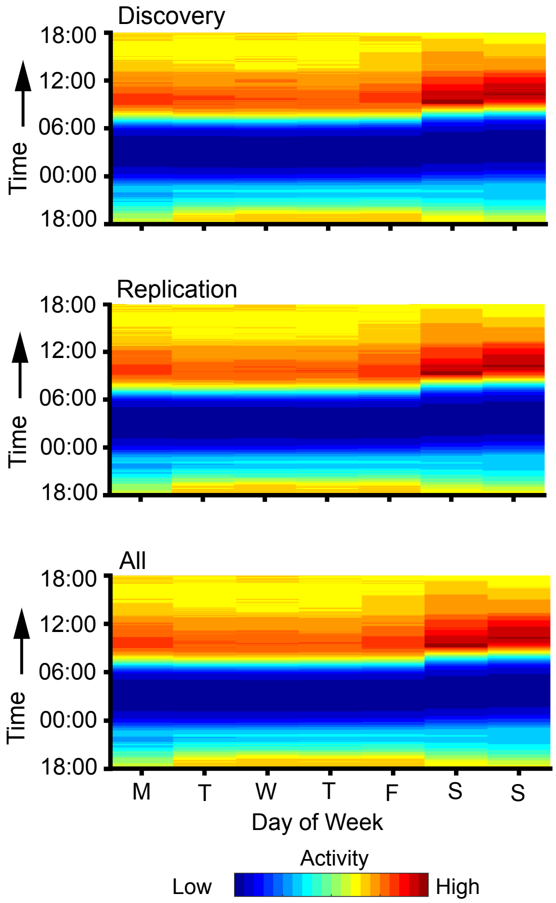

Supp. Figure 5

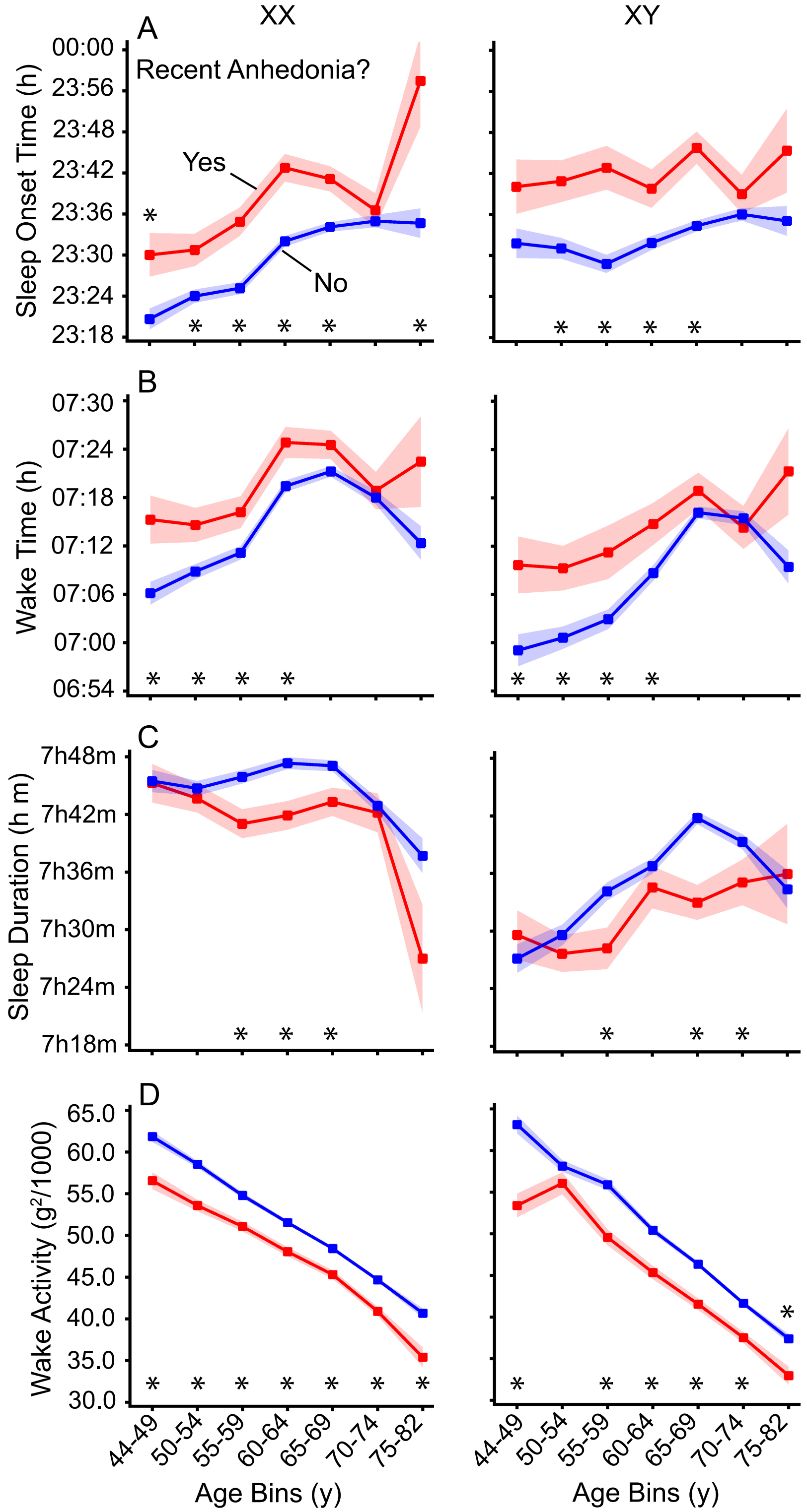

Supp. Figure 6

Tables

Supp. Table I. Sleep Parameters Difference: Results of independent samples *t*-tests examining sex differences in sleep parameters (sleep onset time, wake time, and sleep duration) across age groups. For each age group, *t*- and *p*-values indicate the magnitude and significance of differences between XX and XY participants.

	**44-49**	**50-54**	**55-59**	**60-64**	**65-69**	**70-74**	**75-82**
**Sleep Onset Time**	t(4782) = -5.46 p<0.001	t(8992) = -5.80 p<0.001	t(10746) = -3.14 p<0.01	t(14281) = 0.18	t(19306) = -1.44	t(13630) = -0.89	t(4782) = -0.81
**Wake Time**	t(4782) = 3.54 p<0.001	t(8992) = 5.32 p<0.001	t(10746) = 6.61 p<0.001	t(14281) = 10.56 p<0.001	t(19306) = 6.58 p<0.001	t(13630) = 3.27 p<0.01	t(4782) = 1.70 p<0.1
**Sleep Duration**	t(4782) = 12.52 p<0.001	t(8992) = 15.04 p<0.001	t(10746) = 12.52 p<0.001	t(14281) = 12.64 p<0.001	t(19306) = 9.37 p<0.001	t(13630) = 4.77 p<0.001	t(4782) = 0.90

Supp. Table II. Wake Activity Difference: Results of independent samples t-tests examining sex differences in wake activity across age groups. t- and p-values are reported for each age group.

	**44-49**	**50-54**	**55-59**	**60-64**	**65-69**	**70-74**	**75-82**
**Wake Activity**	t(4782) = 0.28	t(8992) = -0.46	t(10746) = -0.36	t(14281) = 4.82 p<0.001	t(19306) = 9.32 p<0.001	t(13630) = 10.82 p<0.001	t(4782) = 5.46 p<0.001
